# Testosterone and estradiol predict male calling performance, but not performance-related tradeoffs, in competitive signaling environments in Cope’s gray treefrogs (*Hyla chrysoscelis*)

**DOI:** 10.1007/s00359-026-01807-3

**Published:** 2026-05-07

**Authors:** Alexander T. Baugh, Megan K. Freiler, Liam N. Halstead, Alexandra Kozak, Mark A. Bee

**Affiliations:** 1 Department of Biology, Swarthmore College, Swarthmore, PA 19081 USA; 2 Department of Ecology, Evolution, and Behavior, University of Minnesota-Twin Cities, St Paul, MN 55108 USA; 3 Graduate Program in Neuroscience, University of Minnesota-Twin Cities, St Paul, MN 55108 USA

**Keywords:** Androgen, Corticosterone, Courtship call, Estradiol, Sexual selection, Testosterone

## Abstract

Acoustic signals in anurans are classic models for understanding how endocrine mechanisms regulate courtship behavior. However, our understanding of how steroids influence male calling performance remains limited because most studies examine single hormones or isolated call traits. Here we quantified three plasma steroids—testosterone (T), estradiol (E_2_), and corticosterone (CORT)—from male Cope’s gray treefrogs (*Hyla chrysoscelis*) recorded in natural male–male calling pairs, relating these concentrations to multiple calling components including call rate, duration, and effort. We used principal components analysis to describe a male’s overall calling *performance* and his calling *strategy*, defined here as a male’s allocation along the tradeoff between producing longer calls versus faster call rates at similar overall effort. We analyzed these relationships at the population level and within male pairs using multiple regressions including all three hormones as predictors. At the population level, T and E_2_ positively correlated with calling performance, whereas calling strategy was unrelated to hormones. Within male pairs, differences in T also positively correlated with differences in calling performance. Despite their positive covariance, both T and E_2_ retained independent partial associations with calling performance. CORT showed no association with calling performance or strategy at either level. These results show that gonadal steroid state predicts asymmetries in calling performance between competitors while remaining unrelated to performance-related tradeoffs. By revealing strong positive correlations between gonadal hormones and energetic investment in calling, this study demonstrates how female preferences for high-performing callers can impose sexual selection on endocrine mechanisms regulating courtship behavior.

**Supplementary Information:**

The online version contains supplementary material available at https://doi.org/10.1007/s00359-026-01807-3.

## Introduction

Sexually selected signals rank among the most physiologically demanding behaviors animals perform, requiring sustained energetic investment and exposing signalers to tradeoffs between reproduction, maintenance, and survival (Andersson 1994). This may be particularly true for species in which signaling occurs in highly competitive social environments, such as a lek or chorus, where males signal in close proximity to attract females (e.g., Vehrencamp et al. 1989). Research on acoustic communication in anurans has been especially informative in this regard given the conspicuous nature of frog advertisement calls, the ability to precisely measure and experimentally reproduce calls, the competitiveness of frog breeding choruses, and the numerous demonstrations that fine-scale variation in call structure, timing, and energetic output governs both male–male interactions and female mate choice (reviewed in Gerhardt and Huber 2002; Wells 2007; Narins et al. 2007). Calling is exceptionally costly in anurans: sustained call production elevates metabolic rate several-fold, accelerates depletion of energetic reserves, and is tightly coupled to mating success (Ryan et al. 1983; Taigen and Wells 1985; Emerson and Hess 2001). Across vertebrates, the expression and performance of sexually selected communication signals is tied to endocrine regulation, with steroid hormones rapidly tracking and modulating signaling effort and with signal production imposing measurable metabolic costs (Amorim et al. 2002; Remage-Healey and Bass 2005). The conservation of neuroendocrine systems thus makes the study of hormonal influences on signaling in anurans applicable across vertebrates (Arch and Narins 2009).

In Cope’s gray treefrog (*Hyla chrysoscelis*), and its tetraploid sister species (*Hyla versicolor*), male advertisement calling represents an energetically demanding performance phenotype under sexual selection. Males attract females in dense breeding choruses by producing short calls composed of discrete pulses generated by repeated contractions of the laryngeal and trunk musculature (McLister et al. 1995; Girgenrath and Marsh 1997, 1999). Pulse rate is species specific, and females prefer calls with pulse rates near the population mean (Gerhardt 1991; Ward et al. 2013). The spectral frequencies of pulses are not strongly correlated with body size, and females exhibit weak and inconsistent preferences based on frequency differences (Schrode et al. 2012). The number of pulses per call (hereafter, ‘call duration’), together with call rate, defines a male’s overall calling effort, determined as the product of call duration and rate (Ward et al. 2013; Krueger et al. 2026). A male’s calling behavior is a strong predictor of the metabolic costs of signaling, with longer calls, faster call rates, and higher overall effort reflecting both greater physiological investment and performance capacity (Taigen and Wells 1985; Wells and Taigen 1986; Grafe 1997; McLister 2001; Schwartz and Rahmeyer 2006). Males producing calls with more pulses tend to do so at slower rates and there is also negative genetic covariance between these two components of calling effort (Sullivan and Hinshaw 1992; Gerhardt et al. 1996; Schwartz et al. 2002; Welch et al. 2014; Reichert et al. 2024). As the competitiveness of local signaling environments increases, males strategically add pulses to lengthen their calls and reduce their call rate while maintaining or slightly increasing their overall calling effort (Wells and Taigen 1986; Reichert and Gerhardt 2012). Here we use ‘strategy’ in a narrow, descriptive sense to refer to a male’s allocation of signaling effort across the rate–duration tradeoff, rather than to imply a fully resolved adaptive tactic or optimization rule (cf. Moody and Fuxjager 2025). Female mate choice is biased toward these costly signals: experimental studies show that females have strong directional preferences for males that produce longer calls at faster rates—and thus signal with higher calling effort—and that these preferences persist across realistic acoustic environments and competitive contexts (Gerhardt 1991; Gerhardt et al. 1996, 2000; Bee 2008; Ward et al. 2013; Vélez et al. 2013; Tanner et al. 2017, 2025; Krueger et al. 2026). Importantly, female evaluation of calling effort reflects the integration of information across multiple calls rather than isolated acoustic traits, placing sustained calling effort—rather than fine-scale spectral or temporal variation—at the center of sexual selection in this system (Ward et al. 2013; Tanner et al. 2017; Krueger et al. 2026).

A longstanding hypothesis in behavioral endocrinology is that such energetically demanding sexual signals are regulated by interacting endocrine axes (Moore et al. 2005; O’Bryant and Wilczynski 2010; Toufexis et al. 2014). Gonadal steroids such as testosterone produced by the hypothalamic–pituitary–gonadal (HPG) axis have long been implicated in facilitating male sexual displays by enhancing vocal motor output, neuromuscular performance, and motivational state (Wetzel and Kelley 1983; Wilczynski et al. 2005; Leary and Baugh 2020). In frogs, prior work has linked circulating steroid state to calling behavior and reproductive condition, including associations among androgens, corticosterone, testes mass, and vocal energetics in breeding males, as well as rapid hormone–behavior coupling during sexual interactions (Emerson and Hess 2001; Crocker-Buta and Leary 2018; Baugh 2024; Freiler et al. 2026). Estradiol (E_2_), although historically understudied in males, is increasingly recognized as a biologically relevant component of the male HPG axis (e.g., Freiler et al. 2026). Beyond developmental effects, E_2_ can act acutely on neural and muscular systems, modulating excitability, endurance, and metabolic efficiency—traits directly relevant to sustained calling behavior (Campbell and Febbraio 2001; Balthazart and Ball 2006; Remage-Healey and Joshi 2012). Thus far, however, E_2_ has rarely been examined explicitly in studies of male frog advertisement calling; the few available studies come largely from Xenopus and indicate that estrogens can alter or suppress male vocal behavior (Schmidt 1983; Hoffmann and Kloas 2012a,b). Furthermore, glucocorticoids released by the hypothalamic-pituitary-adrenal/inter-renal axis (HPA/I axis), such as corticosterone (CORT), regulate energy mobilization during periods of high metabolic demand and have been proposed to either facilitate sustained signaling or suppress reproductive effort when energetic reserves are limited. Emerson’s Energetics–Hormone Vocalization (EHV) model integrates these ideas, predicting that prolonged calling elevates glucocorticoids, which may in turn inhibit gonadal steroid production and down-regulate sexual signaling (Emerson and Hess 2001). Subsequent work has provided mixed support for these predictions: some studies report positive or context-dependent links between glucocorticoids and reproductive behavior, whereas others find weak or absent associations between corticosterone and acoustic signaling (e.g., Emerson and Hess 2001; Crocker-Buta and Leary 2018; Leary and Baugh 2020; Baugh 2024). Although influential, empirical support for this framework in anurans has been mixed. For example, Emerson and Hess (2001) reported covariance among corticosterone, androgens, testes mass, and calling energetics in breeding frogs, whereas later work has emphasized bidirectionality and social-context dependence in hormone–behavior relationships and has not always found simple linear relationships between glucocorticoids and signaling effort (Crocker-Buta and Leary 2018; Leary and Baugh 2020). Progress on such questions has been slowed by two major limitations of previous studies. First, most studies rely on analyzing single acoustic traits despite evidence that both female preferences and male performance are inherently multivariate. Second, few studies address the additional challenge that males do not signal in isolation but instead compete against other nearby males to attract females.

Here, we combine paired acoustic recordings of neighboring males in breeding choruses with endocrine sampling in *H. chrysoscelis* to investigate how gonadal and inter-renal hormones relate to male calling behavior in a competitive context. We measured male calling behavior and circulating T, E_2_, and CORT to address two complementary questions. First, do gonadal and inter-renal hormones explain variation in calling behavior across males at the population level? Second, do asymmetries in endocrine state between nearby males competing in a chorus predict asymmetries in calling behavior? By including contrasts of two competing males, we aimed to isolate endocrine effects while controlling for shared environmental, temporal, and acoustic conditions.

## Materials and methods

### Acoustic recordings, blood collection, and biometrics

**Fig. 1:**
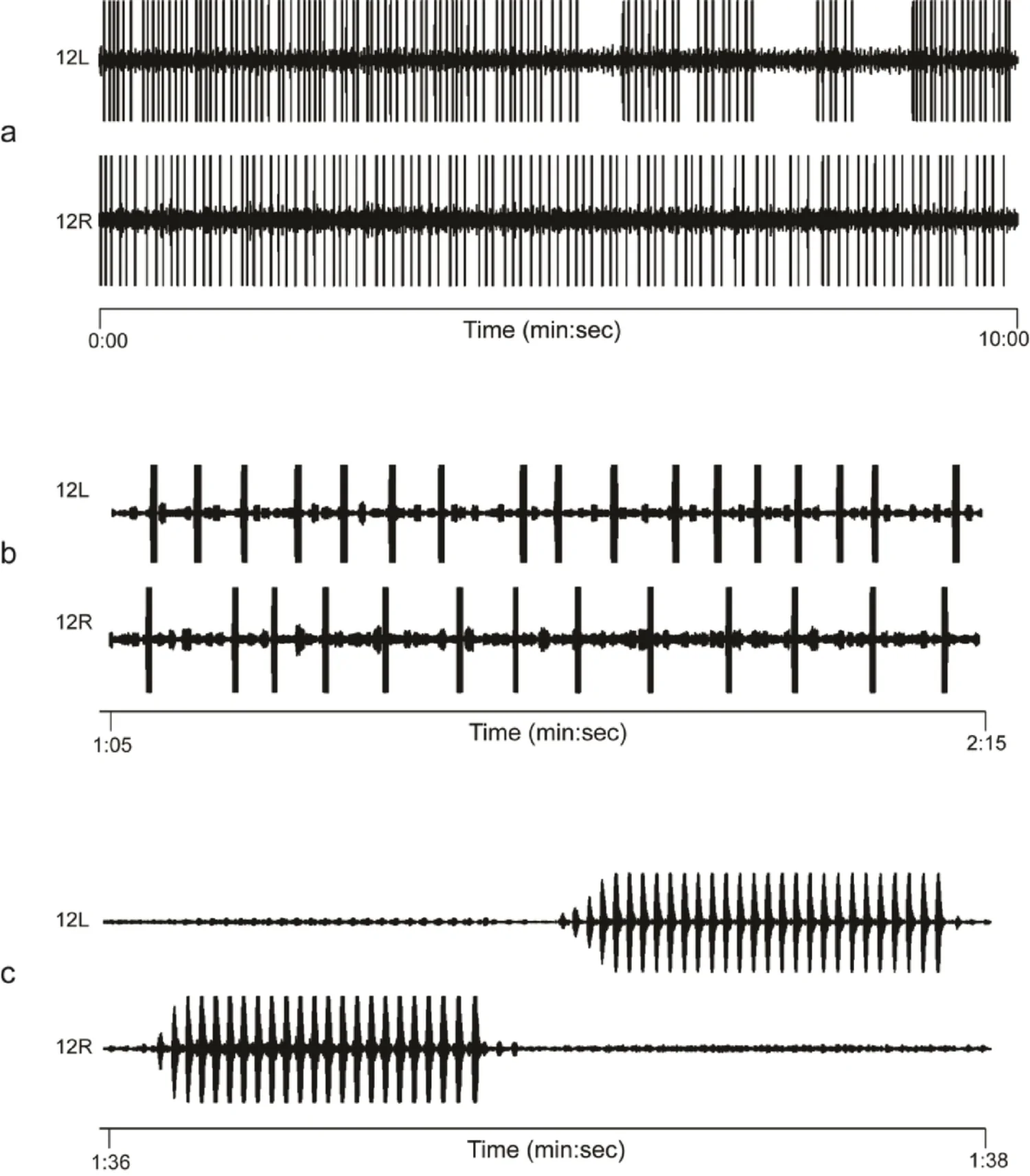
Two-channel recording depicting waveforms of two adjacent and vocally competing *H. chrysoscelis* males (pair 12) recorded on 19 June 2024. The left channel is Frog 12 L and the right channel is Frog 12R. **a** Full 10-min recording. Despite gaps in calling by 12 L, he produced more calls (114) than 12R (101) because of shorter inter-call intervals, and he exhibited substantially higher calling effort (446 pulses min − 1 versus 247 pulses min − 1). **b** Shorter segment of the 10-min recording illustrating differences in call rate between males 12 L and 12R. **c** Sequential calls from 12 L and 12R showing 28 and 23 pulses, respectively

We conducted paired-male recordings of the western genetic lineage of Cope’s gray treefrog (Booker et al. 2022) from wetlands located in the Carver Park Reserve (Carver County, MN, USA, 44° 52′ 33.5″ N, 93° 41′ 03.4″ W ) during peak calling periods (June 10–20, 2024; 2200–2330). Each recording involved two neighboring males calling in close proximity in a breeding chorus, allowing us to quantify variation in calling behavior at the within-pair level in a competitive signaling environment (*n* = 14 pairs). Field procedures followed a standardized protocol performed by a three-person team (audio manager, microphone operator, and one blood-sampling technician). Pairs of males were located visually using red headlamps in shallow water and selected only if (a) both were actively calling, (b) each male within a pair was the other’s nearest calling neighbor (within-pair separation: median = 1.83 m, mean = 3.69 m, SD = 3.29 m), and (c) they were positioned sufficiently apart from neighboring males to isolate each dyad to obtain recordings suitable for analysis (Fig. 1). Directional microphones (Sennheiser ME66; Sennheiser Electronic Corporation, Old Lyme, CT, USA) were placed in front of each male (∼1 m distance), and a 10-min simultaneous recording (Zoom H8, Zoom Corporation, Tokyo, Japan) was initiated (SM1 & SM2).

Immediately after the 10-min recording window, we sampled blood via cardiac puncture following established methods validated in *Hyla* (e.g., Bastien et al. 2018; Baugh 2024). Briefly, we rapidly (ca. 3 min; range = 1–7 min measured to nearest minute) collected blood (ca. 50 µL) using a 30-gauge insulin syringe (BD Micro-fine U-100, 0.3 mL) pre-rinsed with heparin. Whole blood was stored at 4 °C on wet ice for 2–4 h and then centrifuged (7500 RPM for 10 min; Eppendorf 5418 at 8° C). The plasma fraction was stored at − 20 °C for 3 weeks and then shipped on dry ice to Swarthmore College where samples were stored at − 80 °C for 1 month until assayed. Immediately after blood collection, we measured water temperature (Fluke 62 Max + IR thermometer, Everett, WA, USA; accuracy: ±1.0 °C), body mass (to the nearest 0.01 g) and body length (to the nearest 0.01 mm) as snout-vent length (SVL). Body condition estimates can be used as an indicator for energy reserves (Leary and Harris 2013). We calculated a body condition index (hereafter ‘BCI’) by obtaining standardized residual values from a linear regression of SVL on body mass. All procedures were approved by the Institutional Animal Care and Use Committee at the University of Minnesota (#2301–40692 A).

### Acoustic analyses

We analyzed recordings of advertisement calls using Adobe Audition 2025 (Adobe Systems Inc., San Jose, CA, USA). Because our primary hypothesis concerned endocrine predictors of sustained calling performance, we focused on the three call traits known to be determinants of the energetic costs of calling and under strong sexual selection due to female mate choice: call rate (calls min⁻¹), call duration (pulses call⁻¹), and calling effort (pulses min⁻¹). For each recorded call, we measured its period (defined as the interval between the onsets of two successive calls) and call duration (defined as the number of pulses in the call). From these values, we computed an instantaneous call rate (calls min⁻¹) as the reciprocal of each call period, and an instantaneous calling effort (pulses min⁻¹) as the product of the instantaneous call rate and call duration for each call. Then for each male, we averaged values over all the calls he produced during the 10-min recording to determine his mean call rate, call duration, and calling effort (see Fig. 1).

### Hormone analyses

Our hormone analyses followed procedures described in more detail elsewhere (Baugh 2024). Briefly, we used a liquid diethyl-ether double extraction protocol. Following extraction, we froze samples at − 80 °C for 1–2 days or we resuspended immediately (assay buffer, provided by kit) and allowed samples to reconstitute for 12 h at 4 °C. In males, optimal reconstitutions are at 1:10 for CORT and E_2_ and 1:80 for T (Baugh 2024). We estimated steroid concentrations using EIA kits (DetectX^®^ kits, Arbor Assays, Ann Arbor, MI) for 17β-estradiol (E_2_; Cat. No. KB30, Donkey anti-Sheep IgG), testosterone (T; Cat. No. K032, Goat anti-Rabbit IgG), and corticosterone (CORT; Cat. No. K014, Donkey anti-Sheep IgG). We analyzed samples in duplicate following manufacturer’s instructions. We used two duplicate internal standards (a single pooled sample of *H. chrysoscelis* plasma) per plate for calculation of intra-assay coefficients of variation (CV). Detection limits and sensitivities, respectively, were 2.05 pg mL^−1^ and 2.21 pg mL^−1^ for E_2_; 9.92 pg mL^−1^ and 30.6 pg mL^−1^ for T; and 16.9 pg mL^−1^ and 18.6 pg mL^−1^ for CORT. Cross-reactivity was 100% for E_2_, 3.2% for estrone sulfate, and 2.5% for estrone; 100% for T, 3.53% for 5a-dihydrotestosterone, and 0.27% for androstenedione; 100% for CORT, 12.3% for desoxycorticosterone, 0.62% for aldosterone, 0.38% for cortisol. Average intra-assay variations were 4.2% for E_2_, 9.3% for T, and 2.6% for CORT.

### Statistical analyses

We assessed the ability of circulating hormones to predict male calling behavior using linear regression to address endocrine effects at both the population level and at the level of adjacent competing males. Hormone concentrations were log₁₀-transformed to improve normality of residuals and then standardized (*z*-scored). For each of our three acoustic measures of calling behavior (call rate, call duration, and calling effort), we standardized (*z*) the individual means for each variable across males and then used principal component analyses (PCA) to reduce the acoustic data to a smaller set of orthogonal variables. Across all three PCA implementations, PC1 consistently loaded positively on all three call-effort traits, whereas PC2 captured a rate–complexity trade-off, confirming stable biological interpretation despite minor sample size differences.

To provide descriptive context for the multiple regressions, we calculated Pearson correlations between hormones, among our three measures of calling behavior, and between hormones and calling behavior using the population-level dataset with complete data (*n* = 24 males). To assess whether BCI or water temperature explained additional variation in calling beyond endocrine predictors, we conducted a series of nested model comparisons within the population-level framework. We compared a base model including standardized log_10_T, log_10_E_2_, and log_10_CORT to models additionally including BCI, water temperature, or both covariates. Model comparisons were evaluated using partial F-tests and Akaike’s Information Criterion (AIC). All analyses were conducted in R (v. 4.4.2). Significance was assessed at α = 0.05, and effect sizes are reported alongside test statistics where appropriate.

## Results

### Calling behavior

On average, males produced advertisement calls consisting of about 28 pulses at a rate of about 12 calls per minute, yielding an average calling effort of about 337 pulses min^-1^ (Fig. 1; Table 1). These values are close to population averages reported in an earlier study of the same population (Ward et al. 2013). Across individuals, mean calling effort varied by a factor of more than 4.0 (range: 120 to 507 pulses min^-1^). Males within pairs also varied in calling (see Fig. 1). As expected, there were significant positive correlations between calling effort and both call rate and call duration, which themselves were negatively correlated (Table 1). Thus, we observed the phenotypic tradeoff between call rate and call duration reported in previous studies.

**Table 1:** Summary statistics, Pearson correlations, and principal component loadings for acoustic variables. The mean ± SD values (*n* = 27) for the three acoustic variables are shown along the diagonal along with the Pearson product moment correlations (r) between variables and their associated p values (two-tailed). Also shown are the factor loadings for the first and second principal components (PC1 and PC2) along with their associated eigenvalues and variance explained

Variable	Calling effort	Call rate	Call duration	PC1	PC2
Calling effort	336.9 ± 81.2 pulses min^− 1^			1.00	0.06
Call rate	*r* = 0.53 *p =* 0.005	12.2 ± 2.7 calls min^− 1^		0.48	0.87
Call duration	*r* = 0.56 *p =* 0.003	*r* = -0.39 *p* = 0.044	28.1 ± 6.1 pulses call^− 1^	0.61	− 0.79
Eigenvalue				1.60	1.40
Variance explained				53.2%	46.4%

A PCA of standardized call traits (*n* = 27 males with complete calling data) yielded two informative axes that together explained 99.6% of the variance (Table 1). PC1 explained 53.2% of the variance and loaded positively on all three acoustic variables, with the heaviest loading on calling effort. Thus, PC1 captured variation in well-established metrics of calling *performance* in gray treefrogs (i.e., higher call rates, longer calls, and higher calling effort). For use in regression analyses, values for PC1 were multiplied by − 1 so that higher scores represent greater overall calling performance. PC2 explained 46.4% of the variance and primarily contrasted call rate (0.89) against call duration (− 0.81), with minimal contribution of calling effort (0.06). We interpret PC2 as capturing orthogonal variation in call allocation along a rate–duration tradeoff: some males produced many short calls whereas others produced fewer longer calls at similar overall calling effort. We refer to this descriptive allocation axis as calling strategy, without implying that it necessarily reflects a discrete or optimal tactic.

### Hormones

Average concentrations for each hormone (Table 2) were typical of patterns observed in vocally active males in these populations (Baugh 2024; Freiler et al. 2026). Circulating T showed the greatest variation among males (coefficient of variation, CV = 1.6), whereas E_2_ varied over a much narrower range (CV = 0.17), and CORT showed intermediate variability (CV = 0.69).

**Table 2:** Summary statistics and Pearson correlations for plasma hormone concentrations. The mean ± SD values (*n* = 24) for the three hormones are shown along the diagonal along with the Pearson product moment correlations (r) between variables and their associated p values (two-tailed)

Variable	Testosterone	Estradiol	CORT
Testosterone (CV = 1.6)	10.0 ± 16.6 ng mL^− 1^		
Estradiol (CV = 0.17)	*r* = 0.39 *p =* 0.057	206.9 ± 36.3 pg mL^− 1^	
CORT (CV = 0.69)	*r* = 0.17 *p =* 0.420	*r* = 0.28 *p* = 0.189	1.37 ± 0.98 ng mL^− 1^

### Hormone-behavior relationships in the population

Pearson correlations between each hormone and each call parameter indicated that gonadal hormones were positively correlated with metrics of calling behavior, especially calling effort (SM3). We used multiple linear regression to test whether circulating hormones predicted variation in calling behavior across males at the population level. We included all males with complete data for all three calling metrics and all three hormones (*n* = 24 males). We calculated PC scores from this subset of males which yielded a component structure consistent (SM4) with the PCA used earlier (see Calling behavior). We then fit linear multiple regression models predicting PC1 and PC2 from standardized (z scores) values of log_10_T, log_10_E_2_, and log_10_CORT:

$$ {\mathrm{PC}} = \upbeta _{0} + \upbeta _{1} ({\mathrm{T}}) + \upbeta _{2} ({\mathrm{E}}_{{\mathrm{2}}} ) + \upbeta _{3} {\mathrm{(CORT)}} + \upvarepsilon $$

**Fig. 2:**
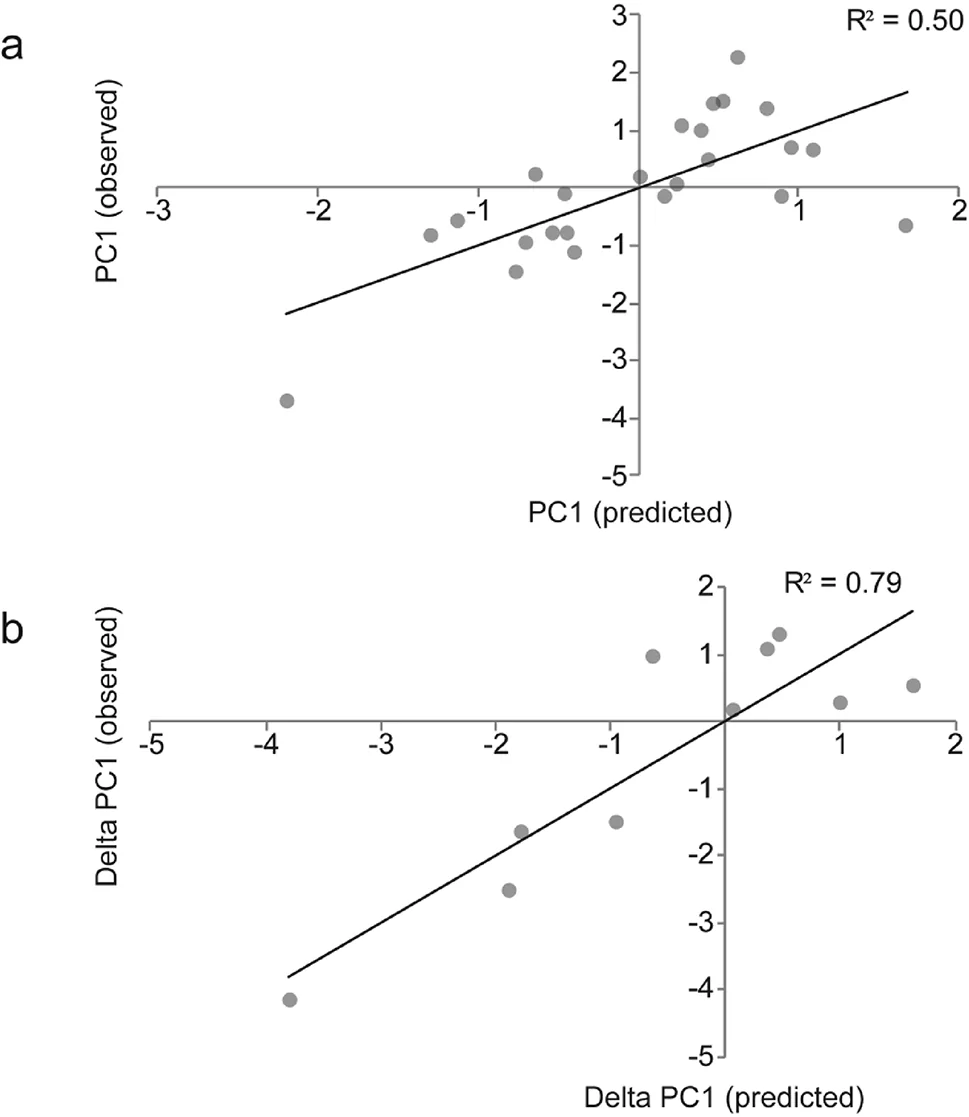
Observed values plotted against values predicted by multiple regression models including standardized log-testosterone, log-estradiol, and log-corticosterone. **a** Overall calling performance (PC1) at the population level (*n* = 24 males). PC1 represents a composite axis derived from call rate, call duration, and calling effort, with higher values indicating greater calling performance. Points represent individual males. **b** Within-pair differences in calling performance (ΔPC1) for competing males (*n* = 10 pairs). Positive values indicate that the male with the higher hormone concentrations exhibited higher calling performance. Predicted values in both panels reflect the combined effects of all three hormone predictors estimated simultaneously

This model was significant for PC1 and revealed that circulating hormones explained substantial variation in overall calling performance at the population level (*R*² = 0.50, adjusted *R*² = 0.42; F_3,20_ = 6.65, *p* = 0.003; Fig. 2a; SM5). Both gonadal hormones showed significant, positive partial effects on calling performance (PC1). T was a positive predictor of PC1 when controlling for E_2_ and CORT (β = 0.55 ± 0.21 SE, *t* = 2.58, *p* = 0.018, 95% CI [0.11, 1.00]). Interpreted in biologically meaningful units, a one–standard deviation increase in T was associated with a 0.55 SD increase in overall calling effort (PC1; ~45 pulses min^− 1^). Likewise, E_2_ independently predicted PC1 (β = 0.52 ± 0.22 SE, *t* = 2.37, *p* = 0.028, 95% CI [0.06, 0.98]), and a one SD increase in E_2_ predicted a 0.52 SD increase (~ 42 pulses min^− 1^), indicating that both gonadal steroids contribute similarly sized effects on calling effort at the population level. In contrast, CORT did not predict PC1 (β = −0.09 ± 0.21 SE, *t* = − 0.42, *p* = 0.680). None of the hormones predicted orthogonal variation in calling strategy (PC2). The PC2 regression model was not significant overall (*R*² = 0.10, adjusted *R*² = −0.03; F₃,₂₀ = 0.78, *p* = 0.522), and no individual predictor approached significance (all *p* > 0.173). Water temperature and BCI did not improve model fit and were thus not included (SM6).

### Hormone-behavior relationships between competing males

To test whether these population‑level associations also explain competitive asymmetries, we next examined hormone and calling differences within male pairs. We used multiple regression to test whether hormonal differences between competing males predicted differences in calling behavior. We included all pairs complete with all three calling metrics and all three hormones (*n* = 10 pairs). We calculated PC scores from this set of 10 pairs (20 males) which yielded a component structure consistent with both PCAs used previously (SM4). For each pair, we calculated differences in PC scores and hormone levels between the two paired males (designated L and R):

$$ \Delta {\mathrm{PC}} = {\mathrm{PC}}_{{\mathrm{L}}} - {\mathrm{PC}}_{{\mathrm{R}}} $$

$$ \Delta {\text{Hormone }} = {\text{ Hormone}}_{{\mathrm{L}}} {-}{\text{ Hormone}}_{{\mathrm{R}}} $$

This difference approach controls for shared environmental, temporal, and acoustic conditions within each dyad and thus helps isolate endocrine predictors of relative calling performance. We then fit multiple regression models predicting ΔPC1 and ΔPC2 from ΔT, ΔE_2_, and ΔCORT:

$$ \Delta {\mathrm{PC}} = \upbeta _{{\mathrm{o}}} + \upbeta _{1} (\Delta {\mathrm{T}}) + \upbeta _{2} (\Delta {\mathrm{E}}_{{\mathrm{2}}} ) + \upbeta _{3} (\Delta {\mathrm{CORT}}) + \upvarepsilon $$

This model was significant for PC1 (*R*² = 0.79, adjusted *R*² = 0.68; F_3,6_ = 7.43, *p* = 0.019; Fig. 2b; SM5), indicating differences in hormone levels between competitors strongly predicted differences in calling performance. Differences in T were a strong predictor of performance asymmetries: males with higher T than their opponent had substantially higher calling efforts (β = 1.24 ± 0.41 SE, *t* = 3.06, *p* = 0.022, 95% CI [0.25, 2.24]). Differences in E_2_ showed a positive but non-significant association with ΔPC1 (β = 0.50 ± 0.27 SE, *t* = 1.84, *p* = 0.12), whereas CORT differences did not predict performance asymmetries (β = 0.24 ± 0.36 SE, t = 0.65, *p* = 0.54). As in our population level approach, hormonal asymmetries did not predict differences in calling strategy (ΔPC2). The ΔPC2 regression model was not significant (*R*² = 0.16, adjusted *R*² = −0.25; F₃,₆ = 0.39, *p* = 0.76), and no hormone predictor approached significance (all *p* > 0.40).

## Discussion

### Female preference and selection on endocrine traits

A key insight from decades of anuran bioacoustics is that female frogs attend closely to a male’s calling performance, including call duration and call rate, calling effort, and overall acoustic output, as indicators of competitive ability (reviewed in Gerhardt and Huber 2002; Wells 2007; Narins et al. 2007). Thus, selection should favor endocrine mechanisms that enable males to sustain high-performance calling in the context of ecological and social constraints. Our results provide a physiological complement to this classic sensory and behavioral framework by showing that coordinated gonadal activation strongly predicts variation in calling performance, but not calling strategy, both across males and between direct competitors. If T or E_2_ facilitates sustained motor output or buffers stress-related suppression of signaling, then female preferences for higher calling performance may impose selection on endocrine phenotypes that facilitate sustained calling performance (Leary and Knapp 2014; Leary and Baugh 2020). In this way, female choice for acoustic performance would shape not only signal structure and signaling behavior but also the hormonal architecture underlying male signaling effort.

### Gonadal hormones and calling performance

Regression analyses at the levels of both the population and competing individuals converged on a robust correlation between gonadal activation and male calling performance. At the population level, hormones strongly (adjusted *R*² = 0.42) predicted a multivariate axis of calling performance, indicating that variation in gonadal steroid state is strongly associated with among-male variation in calling performance. A similar relationship was found comparing nearby competing males, where differences in hormones predicted differences in calling performance with an even larger effect size (adjusted *R*² = 0.68). This convergence suggests that the paired design may have increased biological resolution by controlling for shared environmental, temporal, and acoustic conditions within dyads. Hence, contrasts between nearby competing individuals provide a particularly powerful and biologically meaningful approach for linking endocrine state to signaling performance.

The partial effects of both T and E_2_ contributed explanatory power to calling performance, with each hormone remaining a significant predictor when controlling for the other. Testosterone’s positive association with calling performance is consistent with its well-established role in male sexual signaling across vertebrates (Kelley 1986; Emerson 2001; Wilczynski et al. 2005; Adkins-Regan 2005; Leary and Harris 2013). Testosterone can enhance vocal motor output, increase calling motivation, and support the neuromuscular performance required for sustained calling (Wetzel and Kelley 1983; Houck et al. 1996; Leary and Baugh 2020). Androgens may also act centrally to bias motivational state toward reproductive investment (Arch and Narins 2009; Alward et al. 2017) and peripherally to facilitate muscle performance (Fuxjager et al. 2013) and respiratory capacity (Husak and Irschick 2009), thereby scaling overall signaling performance. In this sense, the strong positive association between T and calling performance observed here is consistent with a large body of work linking androgenic state to elevated reproductive effort. The lack of a relationship between hormones and calling strategy—defined here as allocation along the call rate–duration tradeoff rather than total signaling output—suggests that circulating steroid state is more closely associated with overall energetic investment than with how that investment is distributed across calls.

In contrast, the finding that E_2_ explained additional, non-redundant variation is especially notable given that research on endocrine regulation of male sexual signaling in anurans has historically emphasized androgens and, to a lesser extent, glucocorticoids, with estrogens receiving comparatively little attention despite their activity in male frogs (Schmidt 1983; Hoffmann and Kloas 2012a,b). This may reflect the fact that circulating E_2_ is derived primarily from the conversion of androgens in the testes and thus considered incidental rather than a hormone with independent behavioral influence. This pattern mirrors a broader vertebrate literature in which estrogenic effects on male behavior are sometimes subsumed under an androgen-focused framework (Ball and Balthazart 2008; but see Balthazart and Ball 2006). Yet E2 receptors and aromatase are abundantly expressed in anuran brain regions involved in vocal production and auditory processing, including the preoptic area and torus semicircularis (e.g., Kelley 1988; Wilczynski and Ryan 2010), suggesting substantial scope for estrogenic modulation of male signaling from systemic or local aromatization sources. In our view, this neural route is the more likely explanation for the present results. Estradiol is well known across vertebrates to rapidly modulate social behavior, aggression, motivation, and sensorimotor processing via central mechanisms (Balthazart and Ball 2006; Remage-Healey and Bass 2006; Remage-Healey and Joshi 2012). Such effects could plausibly enhance the persistence or intensity of calling without requiring estradiol to act directly on the calling musculature. Although we cannot rule out the possibility that E2 also contributes peripherally to aspects of sustained performance, evidence for strong direct effects of estradiol on muscle relevant to male frog calling is currently limited. We therefore regard central estrogenic modulation of competitive signaling circuits as the more parsimonious interpretation of the present findings. Such central estrogenic effects are especially relevant for prolonged anuran calling, which depends not only on muscular endurance but also on sustained motivation, sensorimotor coordination, and modulation of vocal-motor circuits during social competition (Taigen and Wells 1985; Emerson 2001; Remage-Healey and Bass 2006; Leary and Baugh 2020). Against this background, our finding that E_2_ explains non-redundant variation in male calling performance indicates that estrogenic pathways may play a more direct role in shaping energetically costly advertisement displays than is typically appreciated. Interestingly, experimentally elevated E_2_ in *Xenopus laevis* has been shown to inhibit male courtship calling (Hoffmann and Kloas 2012a), which occurs through estrogen receptor signaling (Hoffmann and Kloas 2012b). A future pharmacological experiment is needed to disentangle the stimulatory and inhibitory roles of T and E_2_ on aspects of male calling.

An additional point requiring explanation is that E_2_ independently predicted calling performance across males at the population level, yet differences in E_2_ between paired callers did not significantly predict performance asymmetries, despite a positive effect estimate. One simple explanation is statistical: the within-pair analysis was based on only 10 dyads and therefore had limited power to detect a moderate estrogenic effect. Biologically, however, the discrepancy may also be informative. Estradiol may better index broader among-male variation in gonadal activation or reproductive state, whereas testosterone asymmetries may more directly map onto short-term competitive differences in signaling output between neighboring males. One possibility is that estrogenic effects act primarily through central circuits involved in motivational or social responsiveness, while androgenic effects more directly influence both central and peripheral components of signal production. Because our calling variables were reduced to multivariate axes, the present data cannot localize the site of action, but the population-versus-dyad contrast suggests that T and E_2_ may not contribute identically to signaling performance.

Future studies will be needed to investigate causal relationships between gonadal hormones and calling performance in gray treefrogs. Experimental manipulations (e.g. elevating, depleting and/or inhibiting T and E_2_) in calling males, ideally combined with measures of metabolic performance, muscle fatigue, and neural activity during sustained calling bouts, would be particularly informative. Integrating such manipulations with paired recordings, as used here, or in experimental contests (e.g., Reichert and Gerhardt 2012) would allow researchers to disentangle hormone-driven changes in signaling capacity from context-dependent motivational effects. Finally, coupling endocrine manipulations of calling males with female phonotaxis assays would provide a powerful test of whether T and E_2_-mediated variation in male calling performance translates into differential mating success, connecting endocrine state, signal production, perception, and selection.

### HPG and HPI interactions

Because T and E_2_ are jointly produced components of HPG-axis activation, linked through aromatization, we speculate that E_2_ could help modulate the extent to which testosterone associated signaling performance is maintained under energetic or stress-related constraints (Wingfield et al. 1990; Remage-Healey and Bass 2006; Handa et al. 2014). Elevated E_2_ may therefore help maintain calling performance under conditions in which activation of the HPI axis would otherwise constrain signaling. This interpretation is consistent with our broader finding that CORT showed little explanatory power for calling performance in this study and suggests that gonadal activation—rather than antagonistic HPG–HPI dynamics—may be the dominant endocrine basis determining calling performance in competitive signaling environments.

The lack of evidence for an influence of CORT on male signaling and the positive correlation observed between circulating gonadal hormones and sexually selected calling performance might help clarify another recent discovery related to energetically demanding sexual behavior in this frog. Specifically, male *H. chrysoscelis* that successfully attract a mate have substantially elevated and correlated plasma hormone concentrations (CORT, androgens and estradiol; Baugh 2024). There are at least two plausible, non-mutually exclusive hypotheses for this pattern: (1) males found in amplexus were chosen by females on the basis of their calling effort, which are positively correlated with gonadal hormone levels (i.e., sexual selection by female choice); or (2) the act of clasping itself rapidly increases steroid hormone secretion (i.e., bidirectionality in hormone-behavior relationships; Crocker-Buta and Leary 2018). The former hypothesis is supported by the current study, and the latter hypothesis is also supported by a recent empirical demonstration that induced amplexus in male *H. chrysoscelis* activates the HPG axis, rapidly elevating both T and E_2_ but not CORT (Freiler et al. 2026). These findings further support the pleiotropic nature of steroids—gonadal steroids are likely facilitating elevated calling performance and thereby potentially increasing mating success (but see Sullivan and Hinshaw 1992) and subsequently becoming further elevated upon clasping to facilitate muscular tone (Kampe and Peters 2013) and sperm release (Silla et al. 2019). The exact role of androgens versus estrogens in these processes remains to be discovered, for example via aromatase inhibition experiments. Lastly, further study of the role of CORT is also needed given its elevated concentrations in naturally but not artificially amplexed males and lack of correlation with calling behavior. For example, the lack of a correlation between endogenous CORT and calling behavior in the present study is in contrast to the experimental effect of exogenous CORT on calling performance in intact male green treefrogs (*Dryophytes cinereus*), where acutely elevated CORT reduces calling effort through decreases in both call duration and call rate (Leary et al. 2021). This pattern of exogenous elevations in CORT influencing sexual behavior despite a lack of a correlation with endogenous levels has also been observed in female mate choice behavior in treefrogs (Baugh et al. 2021) and is a more general feature of hormone-behavior relationships (Goymann and Dávila 2017). Interpreting these effects in intact animals is complicated by potential interactions with negative feedback mechanisms, but further study is needed given evidence that the phase of CORT secretion specifically can impact behavioral responses (Sarabdjitsingh et al. 2010).

Our conclusion that higher concentrations of gonadal steroids, but not CORT, are associated with higher calling performance requires some qualification. The current study used a 10-min sample of calling activity and a single acute blood sample, with the assumption that these two levels of the phenotype are temporally synchronized and representative more generally. However, a 10-min sample of calling activity during a 10-day period of the breeding season represents a narrow slice of time. The potential influence of CORT on calling performance might only be revealed at extreme concentrations or be relevant only at the performance limits of the males (Marler and Ryan 1996; Leary and Knapp 2014). In addition, like other lek-breeding frogs (Ryan 1985), male gray treefrogs can spend several hours per night, across many nights, calling (Sullivan and Hinshaw 1992; Runkle et al. 1994; Wells et al. 1995; Gerhardt et al. 1996; Reichert et al. 2024). How males trade-off calling performance in a given bout against performance distributed across an entire night, and across an entire breeding season is not yet well understood. Further, how hormones might provide a mechanistic basis for resolving these temporal-investment tradeoffs is unknown. However, this tradeoff should be important in principle; for example, Sullivan and Hinshaw (1992) demonstrated that the single best predictor of male mating success in the tetraploid gray treefrog, *H. versicolor*, is the number of nights a male participates in the chorus, a pattern also observed in túngara frogs (Ryan 1985). Therefore, understanding how males optimize the distribution of vocal effort across different time scales, how interactions between the HPG and HPI axes influence these decisions, and how behavioral and hormonal phenotypes are linked with ecological variables (e.g., foraging; Green 1990; Marler and Ryan 1996; Wilhite and Ryan 2024; predation risk: Baugh and Ryan 2010) and social factors (e.g., female cues; Akre and Ryan 2011) represent fruitful directions for further study.

## Conclusion

By linking variation in calling performance to underlying endocrine state in both population-level and paired competitive contexts, the present study provides evidence of physiological mechanisms that subserve male performance in competitive signaling environments. In this regard, this study builds on the enormous body of work by the honoree of this special issue, Peter Narins. A fundamental theme running through Narins’ research has been to understand anuran signaling behaviors in competitive contexts, and this research has profoundly shaped our understanding of acoustic competition among male frogs and its consequences for sexual selection. Most directly related to the present study, Lopez and Narins (1991) demonstrated that females of the Puerto Rican Coqui, *Eleutherodactylus coqui*, strongly prefer males calling at higher rates, an earlier finding that helped establish the fundamental importance of female preferences for male calling performance. To better understand how males achieve such performance under competitive conditions, Narins and colleagues have examined the interactions among calling males, often using the evoked vocal response as a powerful assay to reveal dynamic signaling strategies. For example, Narins and Capranica (1978) identified the functional role of the “Co” note in competitive signaling among Coqui males, Lopez and Narins (1988) documented striking plasticity in call intensity and frequency, and Benedix and Narins (1999) found robust call-rate escalation during simulated intrusions. Narins’ work has also investigated how males cope with the cacophony that results when competing males call in noisy chorus environments. For example, Zelick and Narins (1983) and Brush and Narins (1989) showed that males precisely time calls to exploit silent windows and avoid call overlap with rival males, while Lewis et al. (2001) revealed the use of seismic signals when acoustic channels were masked. Finally, Narins has also shown that vocal interactions extend beyond considerations of the form and timing of acoustic signals by showing that physical aggression between competing males depends on multimodal cues and cross-modal integration during territorial defense (Narins et al. 2003, 2005). Together, these and other pioneering studies by Peter Narins and his students, postdocs, and collaborators help frame our endocrine findings within a rich tradition of neuroethological research on the behavioral strategies that enable male success in competitive signaling environments.

## Supplementary Information

Below is the link to the electronic supplementary material.

Supplementary Material 1

Supplementary Material 2

Supplementary Material 3

Supplementary Material 4

Supplementary Material 5

Supplementary Material 6

Supplementary Material 7

## Data Availability

Complete dataset available in SM7.
