# Supplementary material for: Testosterone and estradiol predict male calling performance, but not performance-related tradeoffs, in competitive signaling environments in Cope’s gray treefrogs (Hyla chrysoscelis)
Source: J Comp Physiol A Neuroethol Sens Neural Behav Physiol. 2026 May 7;212(5):753–64. doi: 10.1007/s00359-026-01807-3 (PMC13642399; doi:10.1007/s00359-026-01807-3)
Supplement: Supplementary file 1 [file 359_2026_1807_MOESM3_ESM.docx]

| Variable 1 | Variable 2 | r | p | N |
| --- | --- | --- | --- | --- |
| log Testosterone | Call rate | 0.21 | 0.323 | 24 |
| log Testosterone | Pulses per call | 0.39 | *0.060* | 24 |
| log Testosterone | Pulses per minute | 0.60 | **0.0019** | 24 |
| log Estradiol | Call rate | -0.02 | 0.921 | 24 |
| log Estradiol | Pulses per call | 0.57 | **0.0039** | 24 |
| log Estradiol | Pulses per minute | 0.52 | **0.0095** | 24 |
| log Corticosterone | Call rate | 0.16 | 0.464 | 24 |
| log Corticosterone | Pulses per call | -0.01 | 0.962 | 24 |
| log Corticosterone | Pulses per minute | 0.15 | 0.481 | 24 |

**SM3:** Pearson correlations were calculated using the population-level dataset (N = 24 males; two-tailed) after excluding non-calling and incomplete individuals. Hormones were log₁₀-transformed prior to analysis.
