# Supplementary material for: Testosterone and estradiol predict male calling performance, but not performance-related tradeoffs, in competitive signaling environments in Cope’s gray treefrogs (Hyla chrysoscelis)
Source: J Comp Physiol A Neuroethol Sens Neural Behav Physiol. 2026 May 7;212(5):753–64. doi: 10.1007/s00359-026-01807-3 (PMC13642399; doi:10.1007/s00359-026-01807-3)
Supplement: Supplementary file 2 [file 359_2026_1807_MOESM4_ESM.docx]

SM4: Supplementary Principal Component Analyses

| Variable | PC1_loading | PC2_loading |
| --- | --- | --- |
| Calling effort (pulses/min) | 0.980 | -0.188 |
| Call duration (pulses/call) | 0.692 | 0.720 |
| Call rate (calls/min) | 0.333 | -0.941 |
|  |  |  |
| Component | Eigenvalue | Variance explained |
| PC1 | 1.550 | 51.66% |
| PC2 | 1.439 | 47.95% |

A. PCA from Hormone-behavior relationships in the population

PCA of standardized calling traits (n = 24 males)

Sample definition: Excluded 2L, 8L, 13L, 14L

Standardization: All three calling traits were z-standardized prior to PCA

| Variable | PC1_loading | PC2_loading |
| --- | --- | --- |
| Calling effort (pulses/min) | 0.923 | -0.379 |
| Call duration (pulses/call) | 0.883 | 0.466 |
| Call rate (calls/min) | 0.062 | -0.997 |
|  |  |  |
| Component | Eigenvalue | Variance explained |
| PC1 | 1.636 | 54.52% |
| PC2 | 1.354 | 45.12% |

B. PCA from Hormone-behavior relationships between competing males

PCA of standardized calling traits (n = 20 males from 10 pairs)

Sample definition: Excluded incomplete pairs: 2L/2R, 8L/8R, 13L/13R, 14L/14R

Standardization: All three calling traits were z-standardized prior to PCA

**Note**: Although numerical factor loadings differed modestly across all three PCA runs due to differences in sample composition, the biological interpretation of each principal component was highly conserved. Across all three PCAs, PC1 consistently loaded positively on pulses per minute, pulse number per call, and call rate, identifying a stable axis of **overall calling effort**. In contrast, PC2 consistently captured a **rate–duration trade-off**, loading strongly and oppositely on call rate versus call duration, with minimal contribution from overall effort. Thus, despite minor variation in coefficient magnitudes, the underlying multivariate structure of calling behavior was highly stable across analytical subsets.
