# Supplementary material for: Testosterone and estradiol predict male calling performance, but not performance-related tradeoffs, in competitive signaling environments in Cope’s gray treefrogs (Hyla chrysoscelis)
Source: J Comp Physiol A Neuroethol Sens Neural Behav Physiol. 2026 May 7;212(5):753–64. doi: 10.1007/s00359-026-01807-3 (PMC13642399; doi:10.1007/s00359-026-01807-3)
Supplement: Supplementary file 3 [file 359_2026_1807_MOESM5_ESM.docx]

**SM5: Commonality** **analyses**

| Variance component | *R*² contribution | Percent of total *R*² |
| --- | --- | --- |
| Unique Testosterone (T) | 0.214 | 42.9 |
| Unique Estradiol (E_2_) | 0.128 | 25.6 |
| Unique Corticosterone (CORT) | 0.003 | 0.6 |
| Shared T + E_2_ | 0.119 | 23.8 |
| Shared T + CORT | 0.002 | 0.4 |
| Shared E_2_ + CORT | 0.001 | 0.2 |
| Shared T + E_2_ + CORT | 0.033 | 6.5 |
| Total model *R*² | 0.499 | 100.0 |

**A.** Commonality analysis for population-level calling performance

Response: PC1_call
Predictors: z_logT, z_logE_2_, z_logCORT
*n*= 24 males

**Interpretation**: Most variance in calling effort was explained by **unique gonadal hormone effects** (T + E2 = 68.5% of total *R*²), with a smaller but meaningful portion attributable to their shared covariance. Corticosterone contributed negligible unique or shared variance.

| Variance component | *R*² contribution | Percent of total *R*² |
| --- | --- | --- |
| Unique ΔTestosterone (ΔT) | 0.454 | 57.6 |
| Unique ΔEstradiol (ΔE_2_) | 0.103 | 13.1 |
| Unique ΔCorticosterone (ΔCORT) | 0.006 | 0.8 |
| Shared ΔT + ΔE_2_ | 0.189 | 24.0 |
| Shared ΔT + ΔCORT | 0.004 | 0.5 |
| Shared ΔE_2_ + ΔCORT | 0.002 | 0.3 |
| Shared ΔT + ΔE_2_ + ΔCORT | 0.040 | 5.1 |
| Total model *R*² | 0.788 | 100.0 |

### B. Commonality analysis for within-pair differences in calling performance

**Response:** ΔPC1_call (male L – male R)
**Predictors:** Δz_logT, Δz_logE_2_, Δz_logCORT **N = 10 pairs**

**Interpretation**: Within pairs, differences in testosterone explained the majority of variance in calling-performance asymmetries, with estradiol contributing both unique variance and shared variance with testosterone. As in population-level analyses, corticosterone contributed negligibly.
