# Supplementary material for: Testosterone and estradiol predict male calling performance, but not performance-related tradeoffs, in competitive signaling environments in Cope’s gray treefrogs (Hyla chrysoscelis)
Source: J Comp Physiol A Neuroethol Sens Neural Behav Physiol. 2026 May 7;212(5):753–64. doi: 10.1007/s00359-026-01807-3 (PMC13642399; doi:10.1007/s00359-026-01807-3)
Supplement: Supplementary file 4 [file 359_2026_1807_MOESM6_ESM.docx]

**SM6: Body condition index (BCI) and water temperature do not explain calling**

Neither body condition nor water temperature explained additional variation in overall calling effort (PC1_call) beyond circulating hormone levels (all partial F-tests p > 0.51; Supplementary Table Sx), and inclusion of these covariates increased model AIC. For call structure (PC2_call), body condition had no effect, whereas water temperature showed a weak association (p = 0.09). However, PC2_call was not predicted by any hormone. Inclusion of temperature did not alter hormone coefficients or qualitative conclusions, and hormone-only models were retained for consistency and parsimony.

| Response | Predictor(s) added | Δdf | F | Pr(>F) | AIC_base | AIC_extended | Interpretation |
| --- | --- | --- | --- | --- | --- | --- | --- |
| PC1_call | Body condition | 1 | 0.036 | 0.851 | 52.27 | 54.22 | No effect |
| PC1_call | Water temperature | 1 | 0.442 | 0.516 | 52.27 | 53.69 | No effect |
| PC1_call | Body condition + temperature | 2 | 0.323 | 0.729 | 52.27 | 55.06 | No effect |
|  |  |  |  |  |  |  |  |
| PC2_call | Body condition | 1 | 0.034 | 0.856 | 72.39 | 74.35 | No effect |
| PC2_call | Water temperature | 1 | 3.119 | 0.096 | 72.39 | 70.65 | No effect |
| PC2_call | Body condition + temperature | 2 | 2.820 | 0.091 | 72.39 | 69.69 | No effect |
